# Glomerular Hyperfiltration Interacts With Abnormal Metabolism to Enhance Arterial Stiffness in Middle-Aged and Elderly People

**DOI:** 10.3389/fmed.2021.732413

**Published:** 2021-10-21

**Authors:** Qi Zhai, Jing Wen, Meiping Wang, Yingting Zuo, Xin Su, Yibo Zhang, Herbert Gaisano, Yan He

**Affiliations:** ^1^Department of Epidemiology and Health Statistics, School of Public Health, Capital Medical University, Beijing, China; ^2^School of Public Health, Baotou Medical College, Baotou, China; ^3^Departments of Medicine and Physiology, University of Toronto, Toronto, ON, Canada; ^4^Municipal Key Laboratory of Clinical Epidemiology, Beijing, China

**Keywords:** arterial stiffness, glomerular hyperfiltration, brachial-ankle pulse wave velocity, pulse pressure, abnormal metabolism

## Abstract

**Introduction:** Glomerular hyperfiltration (GHF) is an early kidney injury. We investigated whether GHF is associated with arterial stiffness expressed by increase of brachial–ankle pulse wave velocity (baPWV) and pulse pressure (PP), and whether the coexistence of GHF and abnormal metabolism increases the risk of arterial stiffness.

**Methods:** In this prospective cohort study, 2,133 non-chronic kidney disease (CKD) participants aged ≥40 years were followed for a mean period of 3.3 years. The extent of arterial stiffness was expressed by measures of baPWV and PP. GHF was defined as eGFR exceeding the age- and sex-specific 90th percentile. Multivariate logistic regression models were used to assess the association between GHF/abnormal metabolism and increased baPWV/PP. The interaction indexes of GHF and abnormal metabolism on arterial stiffness were calculated based on the OR in a multivariate logistic regression model.

**Results:** GHF alone was not associated with increased baPWV or PP in all participants in this study. However, when GHF coexisted with abnormal metabolism, the risk of increased PP increased 3.23-fold [OR = 3.23(1.47–7.13)] compared with participants with normal filtration and normal metabolism, in which the interaction accounted for 55.1% of the total effect and 79.8% of the effect from GHF and abnormal metabolism. After subtracting the independent effects of GHF and abnormal metabolism, their combined effect still resulted in a 1.78-fold increase in PP.

**Conclusion:** GHF could interact with abnormal metabolism to significantly enhance arterial stiffness. Since abnormal metabolism commonly exists in the general population, even slight changes in renal function should be distinguished to prevent arterial stiffness risk.

## Introduction

Chronic kidney disease (CKD) is an identified risk factor for cardiovascular diseases (CVDs) and independent of traditional CVD risk factors (1). Decline of eGFR in CKD patients might cause micro- and macrovascular disease through arterial stiffness, which is considered as a possible mechanism of cardiorenal connection (2). GHF is an intermediate process of eGFR from normal to decline. However, whether GHF could increase the risk of arterial stiffness as eGFR decline does is not clear (3, 4).

Molecular studies indicated that the primary pathology causing GHF included activation of the renin angiotensin aldosterone system (RAAS) (5) and sympathetic nervous system (6), as well as oxidative stress and systemic inflammation, which may drive vascular lesions and lead to arterial stiffness (7). On the basis of these studies, we postulated that GHF may cause CVDs by increasing arterial stiffness. Abnormal metabolism is an important risk factor for inflammation, which could also be able to aggravate arterial stiffness (8). Abnormal metabolism is highly prevalent in the general population. However, When GHF and abnormal metabolism coexist, whether they could interact to enhance arterial stiffness is not clear.

Brachial–ankle pulse wave velocity (baPWV) and pulse pressure (PP) are the early clinical manifestation of pathological alteration of blood vessels and CVDs, both of them are indicators of arterial stiffness (9, 10). In this study, we used baPWV and PP as indicators to examine the influence of GHF and abnormal metabolism on arterial stiffness. We also investigated the influence of coexistence of GHF and abnormal metabolism on arterial stiffness.

## Methods

### Study Design and Participants

**Figure 1:**
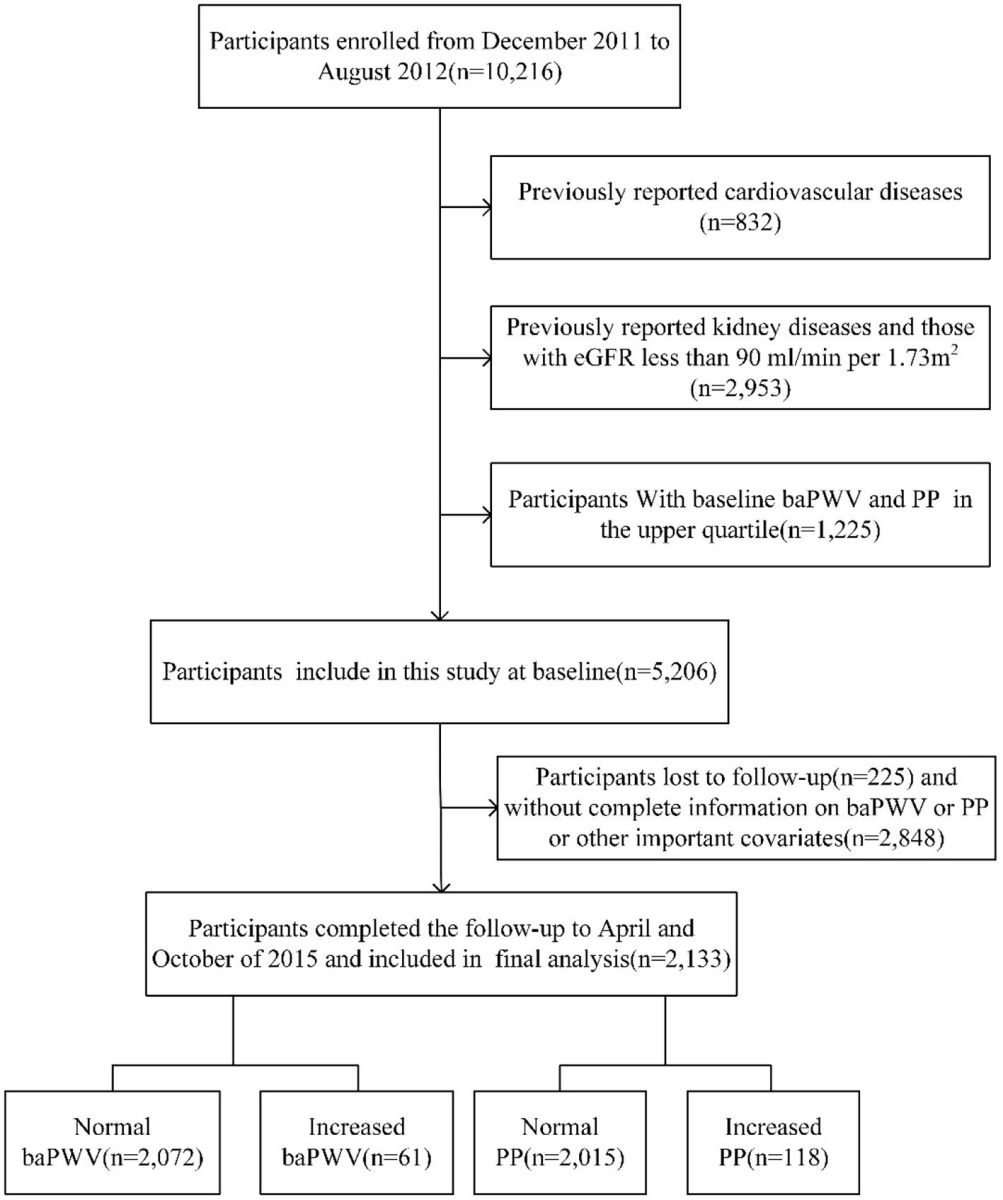
Study flow diagram. baPWV, brachial–ankle pulse wave velocity; PP, pulse pressure; eGFR, estimated glomerular filtration rate.

The survey Risk Evaluation of Cancers in Chinese Diabetic Individuals (REACTION) is an on-going study (11). The purpose of REACTION is to demonstrate the association between diabetes and cancer in the Chinese population. Considering the representativeness of the general population in China, this study selected 25 local communities in different regions of China based on geographic area, degree of urbanization, and economic development. Pingguoyuan communities, Beijing, China, is a single city center in the REACTION study. From December 2011 to August 2012, 10,216 participants aged 40 years or over were enrolled from the Pingguoyuan communities of Beijing and the first follow-up was conducted 3 years later. The flow chart of this study is shown in Figure 1, we excluded the following participants: (1) previous kidney diseases, (2) previous CVDs, including myocardial infarction, stroke, and coronary artery disease, (3) eGFR <90 mL/min/1.73 m^2^, (4) baseline baPWV and PP in the upper quartile, and (5) incomplete information on baPWV or PP or other important covariates. Thus, 2,133 participants were finally included in the analysis.

This study was approved by the Medical Ethics Committee of Ruijin Hospital, Shanghai Jiaotong University. All study participants provided written informed consent at baseline in accordance with the Declaration of Helsinki.

### Outcomes

The main outcomes were increased baPWV and increased PP, which were defined as baPWV and PP equal or higher than their respective cut-off point at follow-up. The cut-off points for increased baPWV and increased PP were determined as the baPWV and PP in their respective upper quartile at baseline (12, 13); in this study they were 1705.13 cm/s and 63.67 mmHg, respectively.

### Data Collection

Collecting baseline information such as demographics, disease history, situation of medication, and behavioral information (smoking and drinking) of participants was undertaken using a standard questionnaire through personal interviews. Height and weight measurements were taken under standard conditions. Body mass index (BMI) was calculated as weight (kilograms) divided by height (meters) squared (kg/m^2^).

PP, baPWV, ankle brachial index (ABI), and heart rate (HR) were measured simultaneously by an automatic waveform analyzer (model VP-1000; Colin Co, Komaki, Japan). The baPWV value was calculated as the distance/transit time ratio (cm/s). The mean of the left-side and right-side baPWV/ABI were obtained for all participants. HR and blood pressure (BP) were calculated as the mean value of the three readings. PP was calculated as systolic blood pressure (SBP) minus diastolic blood pressure (DBP).

A total of 10 mL of blood samples was provided by all participants after an overnight fast (at least 10 h) for biochemical analyses, including fasting plasma glucose (FPG), glycated hemoglobin A_1c_ (HbA_1c_), total cholesterol (TC), triglycerides (TG), high-density lipoprotein cholesterol (HDL-C), low-density lipoprotein cholesterol (LDL-C), and serum creatinine (Scr). Lipids and Scr were measured with an autoanalyzer (c16000 system, ARCHITECT ci16200 analyzer; Abbott Laboratories, Chicago, IL, USA), in which the creatinine measurements were standardized to an isotope dilution mass spectrometry reference measurement procedure.

### Definitions

GHF was defined as an eGFR over the 90th percentile in different age (40–49, 50–59, and ≥60 years) and sex categories (14) at baseline, the 90th percentile cut-off points (15–17) of the Modification of Diet in Renal study (MDRD) eGFR were from 124.65 to 149.58 mL/min per 1.73 m^2^.

Three main metabolic risk factors including plasma glucose, BP, and lipids were used to define the population with abnormal metabolism (18). According to baseline data, dysglycemia was defined by the ADA criteria (19) or self-reported previous diagnosis of diabetes mellitus (DM) by physicians or taking antidiabetic medications. Hypertension was defined as use of any antihypertensive agents and/or SBP ≥ 140 mm Hg and/or DBP ≥ 90 mm Hg (20) or having been previously diagnosed by a health professional. Dyslipidemia was defined as TC ≥ 6.22 mmol/L and/or TG ≥ 2.26 mmol/L and/or LDL-C ≥ 4.14 mmol/L and/or HDL-C <1.04 mmol/L according to Chinese guidelines on prevention and treatment of dyslipidemia in adults (21).

Risk factors such as current smoking and drinking were adjusted as covariates. Participants who smoked one cigarette per day or seven per week regularly during the past 6 months were defined as current smokers. The frequency of alcohol consumption was recorded, and those who consumed alcohol once per week regularly during the past 6 months were defined as current drinkers.

At the end of the follow-up, new onset DM was defined as no dysglycemia at baseline but diagnosed as DM at follow-up. New onset hypertension, dyslipidemia, and overweight/obesity were defined as no hypertension, dyslipidemia, and overweight/obesity at baseline but diagnosed at follow-up, respectively. Being overweight was defined as a BMI of 25.0–29.9 kg/m^2^ and obesity was defined as a BMI of 30.0 kg/m^2^ or higher, according to the World Health Organization definitions.

### Statistical Analysis

All statistical analyses were performed with SPSS software V.23.0 for Windows (SPSS, Chicago, IL, USA). Continuous variables were described as means ± SD or medians (inter-quartile ranges). Categorical variables were described using percentages. Differences in laboratory values between groups were evaluated using Student's *t* test for normally distributed continuous data, Kruskal–Wallis test for skew-distributed continuous data, or χ^2^ test for categorical variables. Except for SBP, because of the skewed distribution of continuous covariates, log-transformation was performed before statistical analysis. ORs and 95% CIs from multivariate logistic regression models were used to assess the association of GHF/abnormal metabolism in all participants with the occurrence of increased baPWV/PP, in which some potential confounding factors were adjusted. The interaction between GHF and abnormal metabolism was evaluated by the synergy index (S), the attributable proportion of interaction (API), the pure attributable proportion of interaction (API'), and the relative excess risk due to interaction (RERI), based on the ORs in the adjusted multivariate regression model (22). Finally, sensitivity analysis was conducted (1) in participants with ABI ≥ 0.9 to avoid the influence of peripheral arterial diseases, (2) to use the 95th percentile (16) cut-off points in different age and sex categories to define GHF, (3) to add baseline baPWV and PP in the covariates, (4) to analyze the association between different GHF status and increased baPWV/PP, and adjust the baseline baPWV and PP, (5) in participants with baseline high baPWV and PP, and adjust the baseline baPWV and PP, and (6) to analyze GHF, abnormal metabolism, and baPWV/PP at follow-up in the generalized linear model in all participants, and to adjust the baseline baPWV and PP.

The statistical tests were two-sided, and a *P* value < 0.05 was considered statistically significant.

## Results

### Characteristics of the Participants

The mean age of the 2,133 participants was 55 (48–57) years and 556 participants (26.1%) were men. Among all participants, 210 participants had GHF. Table 1 shows the baseline characteristics of participants stratified by GHF. Baseline PP in participants with GHF was higher than those without GHF.

**Table 1:** Characteristics of individuals with normal glomerular filtration or hyperfiltration.

**Characteristics**	**Total participant**	**Normal filtration**	**Hyperfiltration**	***p* ^**a**^**
	**(*n* = 2,133)**	**(*n* = 1,923)**	**(*n* = 210)**	
**Baseline**				
Age (years)	53 (48–57)	53 (48–57)	52 (48–55)	0.26
Male, No. (%)	556 (26.1)	502 (26.1)	54 (25.7)	0.903
BMI (kg/m^2^)	25.3 (23.2–27.5)	25.3 (23.2–27.5)	25.5 (23.7–27.6)	0.101
Current smokers, No. (%)	317 (14.9)	283 (14.7)	34 (16.2)	0.738
Current drinkers, No. (%)	226 (10.6)	203 (10.6)	23 (11)	0.649
eGFR (mL/min/1.73 m^2^)	114.1 (103.4–127.3)	112.1 (102.3–122.9)	151.5 (144.7–161.7)	<0.001
TC (mmol/L)	5.3 (4.7–5.9)	5.3 (4.7–5.9)	5.1 (4.6–5.8)	0.119
TG (mmol/L)	1.2 (0.9–1.7)	1.2 (0.9–1.7)	1.2 (0.8–1.6)	0.438
LDL- C (mmol/L)	3.19 (2.7–3.7)	3.2 (2.7–3.7)	3.2 (2.6–3.7)	0.789
HDL- C (mmol/L)	1.5 (1.2–1.7)	1.5 (1.2–1.7)	1.4 (1.2–1.7)	0.376
HbA_1c_ (%)	5.9 (5.6–6.2)	5.9 (5.6–6.2)	5.9 (5.7–6.3)	0.046
SBP (mm Hg)	124.3 ± 11.3	124.1 ± 11.3	125.6 ± 11.2	0.071
DBP (mm Hg)	74 (68–79.7)	74 (68.3–79.7)	73.7 (67.9–79.3)	0.722
HR (beats/min)	77 (70.7–83.7)	76.7 (70.7–83.7)	77.3 (72–83.4)	0.201
PP (mm Hg)	50.3 (45.2–55.7)	50.3 (45–55.7)	52.5 (47.3–57)	0.009
baPWV (cm/s)	1,400 (1279.8–1,517)	1,398 (1,278–1511.5)	1411.8 (1293.9–1550.8)	0.1
**Follow-up**				
PP (mm Hg)	46 (39.7–52.7)	46 (39.7–52.7)	47 (40–53)	0.259
baPWV (cm/s)	1,290 (1,170–1426.5)	1289.5 (1167.5–1425.5)	1,296 (1174.6–1437.4)	0.983
New onset Hypertension, No. (%)	152 (7.1)	142 (7.4)	10 (4.8)	0.161
New onset Diabetes, No. (%)	103 (4.8)	92 (4.8)	11 (5.2)	0.771
New onset Dyslipidemia, No. (%)	224 (10.5)	205 (10.7)	19 (9)	0.469
New onset Overweight/Obesity, No. (%)	71 (3.3)	62 (3.2)	9 (4.3)	0.415

*BMI, body mass index; TC, total cholesterol; TG, triglyceride; eGFR, estimated glomerular filtration rate; HDL- C, high-density lipoprotein cholesterol; LDL- C, low- density lipoprotein cholesterol; HbA1c, glycated hemoglobin A1c; SBP, systolic blood pressure; DBP, diastolic blood pressure; HR, heart rate; PP, pulse pressure; baPWV, brachial–ankle pulse wave velocity.*

*Data were expressed as median (IQR 25–75%) for continuous variables, except SBP which was expressed as mean ± SD, number (percentage) for categorical variables.*

a *p from t test, Kruskal-Wallis test or chi-square, comparing the normal filtration with the hyperfiltration group*.

### GHF Was Not Associated With Increased baPWV/PP in All Participants

During a median follow-up of 3.3 (IQR, 3.2–3.3) years, 61 and 118 participants developed increased baPWV and increased PP, respectively. The associations between GHF or abnormal metabolism with increased baPWV/PP in all participants were examined in multivariate logistic regression models. After adjusting for some variables as indicated in Table 2, we found that both GHF {baPWV [OR = 1.21 (0.50–2.95)] and PP [OR = 1.73 (0.91–3.30)]} and abnormal metabolism {baPWV [OR = 1.29 (0.68–2.46)] and PP [OR = 1.66 (0.98–2.82)} were not associated with increased baPWV/PP compared with the normal glomerular filtration or normal metabolism, respectively. Abnormal metabolism tended to be a risk factor for increased PP (*P* = 0.059).

**Table 2:** Association between hyperfiltration/abnormal metabolism and outcomes in all participants.

	**Increased baPWV**			**Increased PP**		
	**Case/N (%)**	**OR (95% CI)^**a**^**	***p***	**Case/N (%)**	**OR (95% CI)^**a**^**	***p***
**Kidney function**						
Normal filtration	213/1,923 (11.1)	Reference		187/1,923 (9.7)	Reference	
Hyperfiltration	25/210 (11.9)	1.21 (0.50, 2.95)	0.673	21/210 (10.0)	1.73 (0.91, 3.30)	0.095
**Metabolic status**						
Normal metabolism	14/574 (2.4)	Reference		24/574 (4.2)	Reference	
Abnormal metabolism	47/1,559 (3.0)	1.29(0.68, 2.46)	0.43	94/1,559 (6.0)	1.66 (0.98, 2.82)	0.059

*PP, pulse pressure; baPWV, brachial–ankle pulse wave velocity.*

*^a^OR (odds ratio) and 95% CI (confidence interval) were from multivariate logistic regression.*

*^a^Adjusted sex, age, current smoking, current drinking, BMI, SBP, TC, TG, LDL-C, HR, HbA1c at base line, new onset hypertension, diabetes, dyslipidemia, overweight/obesity during follow-up for kidney function and sex, age, current smoking, current drinking, BMI, HR at base line, New onset hypertension, diabetes, dyslipidemia, overweight/obesity during follow-up for metabolic status*.

### The Coexistence of GHF and Abnormal Metabolism Enhance the Risk of Increased PP

To examine whether GHF and abnormal metabolism interact to influence arterial stiffness, the participants were divided into four groups: A0H0, A1H0, A0H1, A1H1 (A0: normal metabolism; A1: abnormal metabolism; H0: normal filtration; H1: GHF) according to their metabolism and GHF status. As shown in Table 3, compared to the participants with normal metabolism and normal filtration, those who had GHF and abnormal metabolism had a significantly increased risk of increased PP [OR = 3.23 (1.47–7.13)]. There was an interaction between GHF and abnormal metabolism on increased PP (S = 4.96), the interaction accounted for 55.11 and 79.82% of the total effect or effect caused by GHF and abnormal metabolism of increased PP, respectively. When combining the effect of GHF and abnormal metabolism minus their independent effect, there was still a 1.78-fold increase in PP.

**Table 3:** Association between groups of different hyperfiltration and metabolic status and outcomes in all participants.

	**Increased baPWV**			**Increased PP**		
	**Case/*N* (%)**	**OR (95% CI)a**	***p***	**Case/*N* (%)**	**OR (95% CI)a**	***p***
**Variables**						
Sex (male)	20/556 (3.6)	Reference		42/556 (7.6)	Reference	
female	41/1,577 (2.6)	0.40 (0.19, 0.85)	0.017	76/1,577 (4.8)	0.54 (0.28, 1.02)	0.057
Age		1.11 (1.06, 1.16)	<0.001		1.12 (1.08, 1.16)	<0.001
BMI		1 (0.92, 1.08)	0.994		1.10 (1.03, 1.17)	0.003
HR		1.01 (0.99, 1.02)	0.215		0.97 (0.95, 0.99)	0.015
Current smoking (never)	53/1,699 (3.1)	Reference		91/1,699 (5.4)	Reference	
Yes	6/317 (1.9)	0.31 (0.11, 0.91)	0.033	21/317 (6.6)	0.63 (0.30, 1.34)	0.231
Current drinking (never)	43/1439 (3.0)	Reference		78/1439 (5.4)	Reference	
Yes	6/226 (2.7)	0.78 (0.26, 2.30)	0.651	16/226 (7.1)	0.82 (0.38, 1.79)	0.629
New onset Hypertension (no)	45/1,981 (2.3)	Reference		70/1,981 (3.5)	Reference	
Yes	16/152 (10.5)	6.04 (3.20, 11.40)	<0.001	48/152 (31.6)	17.46 (10.86, 28.06)	<0.001
New onset Diabetes (no)	56/2,030 (2.8)	Reference		106/2030 (5.2)	Reference	
Yes	5/103 (4.9)	1.37 (0.50, 3.79)	0.54	12/103 (11.7)	1.55 (0.72, 3.34)	0.261
New onset Dyslipidemia (no)	50/1,909 (2.6)	Reference		107/1,909 (5.6)	Reference	
Yes	11/224 (4.9)	2.23 (1.10, 4.51)	0.026	11/224 (4.9)	0.88 (0.43, 1.80)	0.716
New onset Overweight/Obesity (no)	59/2,062(2.9)	Reference		114/2,062 (5.5)	Reference	
Yes	2/71 (2.8)	0.97 (0.22, 4.33)	0.972	4/71 (5.6)	1.22 (0.38, 3.97)	0.741
**Groups**						
A0H0	13/528 (2.5)	Reference		22/528 (4.2)	Reference	
A1H0	42/1,395 (3)	1.26 (0.65, 2.45)	0.502	81/1,395 (5.8)	1.52 (0.88, 2.65)	0.137
A0H1	1/46 (2.2)	1.07 (0.13, 8.83)	0.953	2/46 (4.3)	0.93 (0.15, 5.68)	0.939
A1H1	5/164 (3)	1.64 (0.55, 4.85)	0.373	13/164 (7.9)	3.23 (1.47, 7.13)	0.004
**Interaction indexes**						
S		1.94			4.96	
API		18.90%			55.11%	
API'		48.44%			79.82%	
RERI		0.31			1.78	

*PP, pulse pressure; baPWV, brachial–ankle pulse wave velocity; S, the synergy index; API, attributable proportion of interaction. API', pure attributable proportion of interaction; RERI, the relative excess risk due to interaction; A0/A1, normal metabolism/abnormal metabolism, H0/H1, normal filtration/hyperfiltration.*

*^a^ OR (odds ratio) and 95% CI (confidence interval) were from multivariate logistic regression, in which sex, age, current smoking, current drinking, BMI, HR at baseline and new onset hypertension, diabetes, dyslipidemia, overweight/obesity during follow-up were adjusted*.

### Sensitivity Analysis

**Figure 2:**
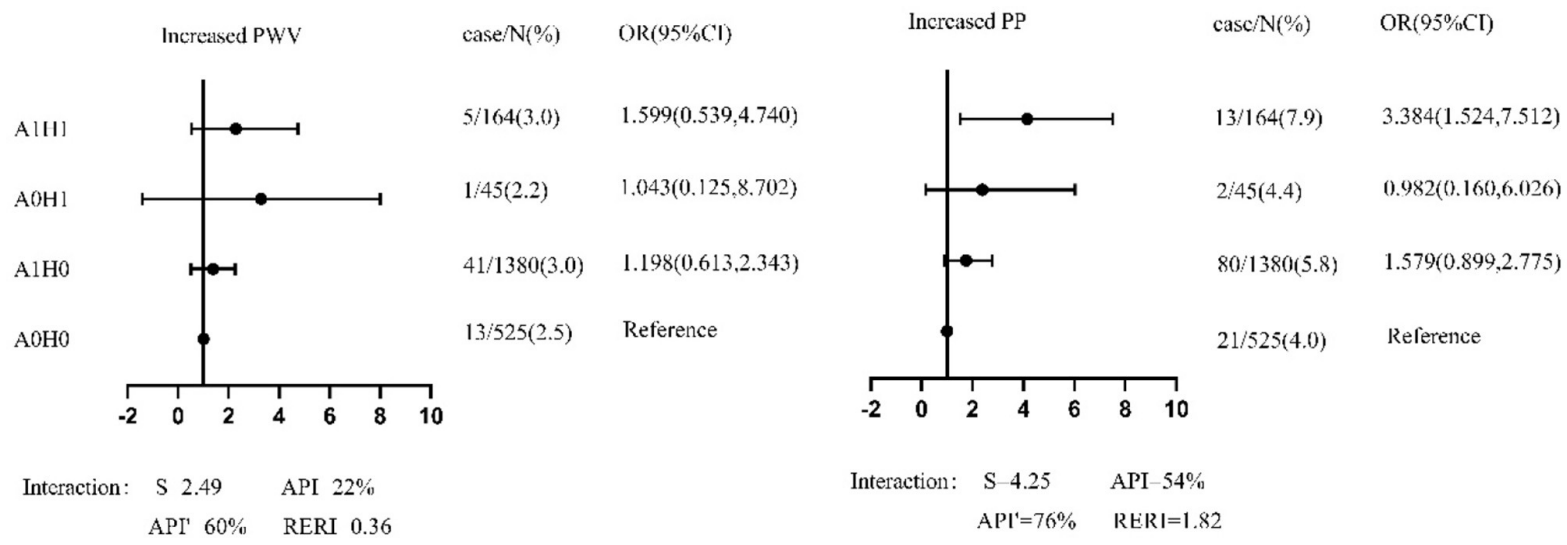
The forest plot of association between groups of different hyperfiltration and metabolic status and outcomes among participants with ABI ≥ 0.9 in sensitivity analysis. PP, pulse pressure; baPWV, brachial–ankle pulse wave velocity; S, the synergy index; API, attributable proportion of interaction. API', pure attributable proportion of interaction; RERI, the relative excess risk due to interaction; A0/A1, normal metabolism/abnormal metabolism; H0/H1, normal filtration/hyperfiltration. OR (odds ratio) and 95% CI (confidence interval) were from multivariate logistic regression, in which sex, age, current smoking, current drinking, BMI, HR at baseline and new onset hypertension, diabetes, dyslipidemia, overweight/obesity during follow-up were adjusted.

In the sensitivity analysis, (1) the 19 participants with ABI <0.9 were excluded to avoid the effects of lower extremity arterial diseases. Figure 2 shows that the coexistence of GHF and abnormal metabolism increased the risk of increased PP [OR=3.38(1.52–7.51)]. The interaction (S = 4.25) accounted for 76% of the risk of increased PP caused by GHF and abnormal metabolism. After subtracting the independent effects of GHF and abnormal metabolism, their combined effect still resulted in a 1.82-fold increase in PP. (2) In Supplementary Tables S1, S2, we define GHF with the 95th percentile in different age and sex categories. Results showed that those with GHF and abnormal metabolism had a significantly increased risk of increased PP [OR = 2.84 (1.034, 7.803)]. After combining the effect of GHF and abnormal metabolism minus their independent effect, there was still a 1.66-fold increase in PP. (3) In Supplementary Tables S3, S4, after adjusting the covariates and baseline baPWV/PP respectively in models, the coexistence of GHF and abnormal metabolism enhanced the risk of increased PP [OR = 2.44 (1.08, 5.49)]. (4) As shown in Supplementary Table S5, those who had GHF at baseline but not at follow-up did not have a statistically significant association with the outcomes. Then all participants were grouped by different GHF status as H00 (normal filtration at baseline and follow-up), H10 (GHF at baseline and normal filtration at follow-up), H01 (normal filtration at baseline and GHF at follow-up), and H11 (GHF at baseline and follow-up). Compared with the H00 group, participants with only baseline GHF, follow-up GHF, and both baseline and follow-up GHF had the trend of increased baPWV and PP; the *P* for all trends was <0.05. (5) In Supplementary Tables S6, S7, baseline high baPWV participants (≥1623.93 cm/s) were referred to as those above the increased baPWV threshold (1705.13cm/s) by a ≤ 5% increase in the baseline level, the same was applied to the definition of baseline high PP (≥60.64 cm/s). GHF increased the risk of increased PP [OR = 4.79 (1.03, 22.36)] in participants with high PP at baseline, the coexistence of GHF and abnormal metabolism significantly enhanced the risk of increased PP [OR = 18.97 (1.80, 200.13)], and even after the combined effect of GHF and abnormal metabolism minus their independent effect, there was still a 15.39-fold increase in PP. (6) As shown in Supplementary Table S8, abnormal metabolism and GHF had an interactive effect and were positively correlated with baPWV [β = 0.016 (0.005, 0.027)] and PP [β = 0.023 (0.007, 0.039)] at follow-up.

## Discussion

In this prospective cohort study, although we found that GHF was not associated with increased baPWV or PP in a 3-year follow-up, the coexistence of GHF and abnormal metabolism significantly increased the risk of increased PP in all participants, and the interaction between GHF and abnormal metabolism accounted for most of this effect.

Some previous cross-sectional studies (23–25) had shown that GHF was not statistically associated with arterial stiffness. However, cross-sectional studies cannot establish causality, and these studies were limited only to CKD or T1 DM participants. The present prospective cohort study was conducted in participants with eGFR ≥ 90 mL/min/1.73 m^2^. Participants with baseline baPWV and PP in the upper quartile were excluded to ensure prospective and further adjusted for the potential risk factors of arterial stiffness, which included new onset diseases or metabolic disorders of DM, hypertension, dyslipidemia, and overweight/obesity during follow-up. Those all made the results more reliable and suggested that GHF was not an independent predictor of arterial stiffness in a period of 3 years. As the earliest stage of kidney injury, GHF and its hemodynamic determinants (the increased glomerular pressures and flows) may injure the capillary network and disrupt normal vascular integrity (26). Therefore, we could not rule out that long-term GHF may increase arterial stiffness and further lead to vascular disease. This view has been confirmed by a prospective study. This study demonstrated that high eGFR was associated with increased risk of CVDs during a 10-year follow-up (27). This association was completely independent of albuminuria, SBP, DBP, and plasma glucose (3). There was also a study suggesting that GHF may be associated with increased arterial stiffness. A cross-sectional study involving participants without CKD (13) suggested that GHF was associated with arterial stiffness measured by baPWV and PP. This study was conducted in participants with high BP and TG, which could significantly enhance the effect of GHF on arterial stiffness as demonstrated by our study, however, this study did not examine whether this effect was from the interaction between GHF and abnormal metabolism or from GHF alone.

Although we found GHF or abnormal metabolism alone was not associated with increased arterial stiffness, we demonstrated that participants with GHF and abnormal metabolism had a 3.23-fold risk of increased PP compared with the participants without GHF or abnormal metabolism; the interaction accounted for 79.8% of the effect. This interaction may partly be explained by some biological pathways or factors that GHF and abnormal metabolism commonly shared, including pathways which may injure the capillary network, large artery dysfunctions (28), and inflammatory factors such as fibrinogen and adhesion molecules, which increase arterial stiffness by causing vascular fibrosis and calcification through collagen deposition (8, 26, 29).

In addition, this study suggested that arterial stiffness caused by GHF and abnormal metabolism was more likely to be reflected by increased PP but not increased baPWV; several studies had observed similar results (30, 31). There are several possible potential mechanisms. On one hand, the common mechanism linking GHF and abnormal metabolism to arterial stiffness through macrovascular dysfunctions may favor an increase in SBP and also a decrease in DBP (28). On the other hand, PWV marked increases with age and out of proportion to the minor increase of PP with age (32); the association between GHF and baPWV may be weakened when analyzing eGFR calculated by age.

This study has several strengths. First, we inferred causality between GHF and arterial stiffness in a prospective cohort study, and discussed the interaction between GHF and abnormal metabolism. Second, we adjusted new onset DM, hypertension, dyslipidemia, and overweight/obesity during follow-up in the analytic model to attenuate their influence on artery stiffness, and some sensitivity analyses were also conducted; it made the results more reliable. There were also several limitations in this study. First, all participants were at age 40 years or over and from a single center only, which could introduce selection bias, but these people could best reflect the benefits of disease prevention. Second, a single measurement of baPWV and BP, rather than two or more measurements over time was used to define arterial stiffness, which may lead to misdiagnosis. Third, GFR was estimated using a single-serum creatinine value entered in the MDRD equation instead of measurement, but this equation is validated in general healthy people individuals with normal kidney function (33). Finally, as the information of proteinuria is not available, the results of association and interaction of this study may biased by the influence of renal injury and its related pathological process which induced proteinuria.

## Conclusions

In this prospective cohort, we found that GHF alone may not be potent enough to enhance arterial stiffness in a relatively short period. However, when GHF and abnormal metabolism coexisted, the unfavorable effect on arterial stiffness significantly increased. The finding from this study emphasizes that future arterial stiffness risks cannot be ignored in subjects with GHF and abnormal metabolism.

## Data Availability Statement

The data in this study were obtained from a third party and are not publicly available. Requests to access these datasets should be directed to jingtaodou@163.com.

## Ethics Statement

This study was approved by the Medical Ethics Committee of Ruijin Hospital, Shanghai Jiaotong University. All study participants provided written informed consent at baseline in accordance with the Declaration of Helsinki.

## Author Contributions

QZ and YH: conception and design and manuscript writing. QZ, YZu, and XS: collection and assembly of data. JW, MW, QZ, YZu, YZh, and XS: data analysis and interpretation. YZh: literature search. HG: editing. YH: review of this manuscript. All authors read and approved the final manuscript.

## Funding

This work was supported by the National Natural Science Foundation of China [Grant Nos: 82073648 and 31672375].

## Conflict of Interest

The authors declare that the research was conducted in the absence of any commercial or financial relationships that could be construed as a potential conflict of interest.

## Publisher's Note

All claims expressed in this article are solely those of the authors and do not necessarily represent those of their affiliated organizations, or those of the publisher, the editors and the reviewers. Any product that may be evaluated in this article, or claim that may be made by its manufacturer, is not guaranteed or endorsed by the publisher.

## References

[1] Piepoli MF Hoes AW Agewall S Albus C Brotons C Catapano AL. 2016 European guidelines on cardiovascular disease prevention in clinical practice. The sixth joint task force of the European Society of Cardiology and Other Societies on Cardiovascular Disease Prevention in clinical practice (constituted by representatives of 10 societies and by invited experts. Developed with the special contribution of the European Association for Cardiovascular Prevention & Rehabilitation. Eur Heart J. (2016) 37:2315–81. 10.1093/eurheartj/ehw106 27222591 PMC4986030

[2] Townsend RR. Arterial stiffness in CKD: a review. Am J Kidney Dis. (2019) 73:240–7. 10.1053/j.ajkd.2018.04.005 29908694 PMC6450550

[3] Mallamaci CZF. The overdriven glomerulus as a cardiovascular risk factor. Kidney Int. (2018) 93:13–5. 10.1016/j.kint.2017.08.035 29291815

[4] Georgianos PI Morfesis P Zebekakis PE. Exploring the association of arterial stiffness with estimated glomerular filtration rate: another chicken-egg paradigm? J Hypertens. (2017) 35:650–1. 10.1097/HJH.0000000000001238 28121844

[5] Cherney DZ Lai V Scholey JW Miller JA Zinman B Reich HN. Effect of direct renin inhibition on renal hemodynamic function, arterial stiffness, and endothelial function in humans with uncomplicated type 1 diabetes: a pilot study. Diabetes Care. (2010) 33:361–5. 10.2337/dc09-1303 19889802 PMC2809283

[6] Rubinger D Backenroth R Sapoznikov D. Sympathetic nervous system function and dysfunction in chronic hemodialysis patients. Semin Dial. (2013) 26:333–43. 10.1111/sdi.12093 23627490

[7] Kanbay M Ertuglu LA Afsar B Ozdogan E Kucuksumer ZS Ortiz A. Renal hyperfiltration defined by high estimated glomerular filtration rate: a risk factor for cardiovascular disease and mortality. Diabetes Obes Metab. (2019) 21:2368–83. 10.1111/dom.13831 31297976

[8] Mozos I Malainer C Horbańczuk J Gug C Stoian D Luca CT. Inflammatory markers for arterial stiffness in cardiovascular diseases. Front Immunol. (2017) 8:1058. 10.3389/fimmu.2017.01058 28912780 PMC5583158

[9] Vlachopoulos C Aznaouridis K Stefanadis C. Prediction of cardiovascular events and all-cause mortality with arterial stiffness. J Am Coll Cardiol. (2010) 55:1318–27. 10.1016/j.jacc.2009.10.061 20338492

[10] Millar JA Lever AF Burke V. Pulse pressure as a risk factor for cardiovascular events in the MRC mild hypertension trial. J Hypertens. (1999) 17:1065–72. 10.1097/00004872-199917080-00004 10466460

[11] Bi Y Lu J Wang W Mu Y Zhao J Liu C. Cohort profile: risk evaluation of cancers in Chinese diabetic individuals: a longitudinal (REACTION) study. J Diabetes. (2014) 6:147–57. 10.1111/1753-0407.12108 24237858

[12] Kim ED Tanaka H Ballew SH Sang Y Heiss G Coresh J. Associations between kidney disease measures and regional pulse wave velocity in a large community-based cohort: the atherosclerosis risk in communities (ARIC) study. Am J Kidney Dis. (2018) 72:682–90. 10.1053/j.ajkd.2018.04.018 30007506

[13] Lin L Peng K Du R Huang X Sun W Ding L. High glomerular filtration rate is associated with arterial stiffness in Chinese population. J Hypertens. (2017) 35:385–91. 10.1097/HJH.0000000000001158 28005707

[14] Cachat F Combescure C Cauderay M Girardin E Chehade H. A systematic review of glomerular hyperfiltration assessment and definition in the medical literature. Clin J Am Soc Nephrol. (2015) 10:382–9. 10.2215/CJN.03080314 25568216 PMC4348676

[15] Melsom T Mathisen UD Ingebretsen OC Jenssen TG Njølstad I Solbu MD. Impaired fasting glucose is associated with renal hyperfiltration in the general population. Diabetes Care. (2011) 34:1546–51. 10.2337/dc11-0235 21593291 PMC3120190

[16] Melsom T Schei J Stefansson VT Solbu MD Jenssen TG Mathisen UD. Prediabetes and risk of glomerular hyperfiltration and albuminuria in the general nondiabetic population: a prospective cohort study. Am J Kidney Dis. (2016) 67:841–50. 10.1053/j.ajkd.2015.10.025 26744126

[17] Lim HI Jun SJ Lee SW. Glomerular hyperfiltration may be a novel risk factor of restrictive spirometry pattern: analysis of the Korea National Health and Nutrition Examination Survey (KNHANES) 2009–2015. PLoS ONE. (2019) 14:e0223050. 10.1371/journal.pone.0223050 31553782 PMC6760802

[18] Grundy SM Stone NJ Bailey AL Beam C Birtcher KK Blumenthal RS. 2018 AHA/ACC/AACVPR/AAPA/ABC/ACPM/ADA/AGS/APhA/ASPC/NLA/PCNA guideline on the management of blood cholesterol: a report of the American College of Cardiology/American Heart Association Task Force on clinical practice guidelines. Circulation. (2019) 139:e1082–143. 10.1161/CIR.0000000000000698 30586774 PMC7403606

[19] American Diabetes Association. Erratum. Classification and diagnosis of diabetes. Sec. 2. In Standards of Medical Care in Diabetes-2016. Diabetes Care 2016;39(Suppl. 1):S13-S22. Diabetes Care. (2016) 39:1653. 10.2337/dc16-er09 27555625

[20] Unger T Borghi C Charchar F Khan NA Poulter NR Prabhakaran D. 2020 International society of hypertension global hypertension practice guidelines. Hypertension. (2020) 75:1334–57. 10.1161/HYPERTENSIONAHA.120.15026 32370572

[21] Chu J Gao R Zhao S Lu G Zhao D Li J. Joint committee for developing chinese guidelines on P, treatment of dyslipidemia in A. Chinese guidelines on prevention and treatment of dyslipidemia in adults. Zhonghua Xin Xue Guan Bing Za Zhi. (2007) 35:390–419. 10.3760/cma.j.issn.0253-3758.2016.10.005 17711682

[22] Hosmer DW Lemeshow S. Confidence interval estimation of interaction. Epidemiology. (1992) 3:452–6. 10.1097/00001648-199209000-00012 1391139

[23] McIntyre NJ Fluck RJ McIntyre CW Fakis A Taal MW. Determinants of arterial stiffness in chronic kidney disease stage 3. PLoS ONE. (2013) 8:e55444. 10.1371/journal.pone.0055444 23383192 PMC3559556

[24] Cherney DZ Sochett EB Lai V Dekker MG Slorach C Scholey JW. Renal hyperfiltration and arterial stiffness in humans with uncomplicated type 1 diabetes. Diabetes Care. (2010) 33:2068–70. 10.2337/dc10-0767 20585001 PMC2928365

[25] Hermans MM Henry R Dekker JM Kooman JP Kostense PJ Nijpels G. Estimated glomerular filtration rate and urinary albumin excretion are independently associated with greater arterial stiffness: the hoorn study. J Am Soc Nephrol. (2007) 18:1942–52. 10.1681/ASN.2006111217 17460143

[26] Hostetter TH Olson JL Rennke HG Venkatachalam MA Brenner BM. Hyperfiltration in remnant nephrons: a potentially adverse response to renal ablation. J Am Soc Nephrol. (2001) 12:1315–25. 10.1681/ASN.V1261315 11373357

[27] Van Biesen W De Bacquer D Verbeke F Delanghe J Lameire N Vanholder R. The glomerular filtration rate in an apparently healthy population and its relation with cardiovascular mortality during 10 years. Eur Heart J. (2007) 28:478–83. 10.1093/eurheartj/ehl455 17223665

[28] Czernichow S Greenfield JR Galan P Jellouli F Safar ME Blacher J. Macrovascular and microvascular dysfunction in the metabolic syndrome. Hypertens Res. (2010) 33:293–7. 10.1038/hr.2009.228 20075933

[29] Czernichow S Greenfield JR Galan P Bastard JP Charnaux N Samaras K. Microvascular dysfunction in healthy insulin-sensitive overweight individuals. J Hypertens. (2010) 28:325–32. 10.1097/HJH.0b013e328333d1fc 20051903

[30] Briet M Bozec E Laurent S Fassot C London GM Jacquot C. Arterial stiffness and enlargement in mild-to-moderate chronic kidney disease. Kidney Int. (2006) 69:350–7. 10.1038/sj.ki.5000047 16408126

[31] Lilitkarntakul P Dhaun N Melville V Kerr D Webb DJ Goddard J. Risk factors for metabolic syndrome independently predict arterial stiffness and endothelial dysfunction in patients with chronic kidney disease and minimal comorbidity. Diabetes Care. (2012) 35:1774–80. 10.2337/dc11-2345 22648437 PMC3402254

[32] Adji A O'Rourke MF Namasivayam M. Arterial stiffness, its assessment, prognostic value, and implications for treatment. Am J Hypertens. (2011) 24:5–17. 10.1038/ajh.2010.192 20940710

[33] Sarwar N Danesh J Eiriksdottir G Sigurdsson G Wareham N Bingham S. Triglycerides and the risk of coronary heart disease: 10 158 incident cases among 262 525 participants in 29 western prospective studies. Circulation. (2007) 115:450–8. 10.1161/CIRCULATIONAHA.106.637793 17190864

