# Supplementary material for: Glomerular Hyperfiltration Interacts With Abnormal Metabolism to Enhance Arterial Stiffness in Middle-Aged and Elderly People
Source: Front Med (Lausanne). 2021 Oct 21;8:732413. doi: 10.3389/fmed.2021.732413 (PMC8566717; doi:10.3389/fmed.2021.732413)
Supplement: Supplementary file 1 [file Data_Sheet_1.docx]

**Table S1.** Association between the 95th percentile cut-off points of hyperfiltration/abnormal metabolism and outcomes in all participants.

|  | Increased baPWV | | |  | Increased PP | | |
| --- | --- | --- | --- | --- | --- | --- | --- |
|  | case/N (%) | OR (95% CI)^a^ | *p* |  | case/N (%) | OR (95% CI)^a^ | *p* |
| *Kidney function* |  |  |  |  |  |  |  |
| Normal filtration | 57/2.31(2.8) | Reference |  |  | 111/2031(5.5) | Reference |  |
| Hyperfiltration | 4/102(3.9) | 1.64(0.56,4..85) | 0.368 |  | 7/102(6.9) | 1.36(0.54,3.38) | 0.515 |
| *Metabolic status* |  |  |  |  |  |  |  |
| Normal metabolism | 14/574(2.4) | Reference |  |  | 24/574(4.2) | Reference |  |
| Abnormal metabolism | 47/1559(3.0) | 0.92(0.48,1.78) | 0.808 |  | 94/1559(6.0) | 1.46(0.84,2.51) | 0.177 |

PP: pulse pressure; baPWV: brachial–ankle pulse wave velocity.

^a^OR (odds ratio) and 95% CI (confidence interval) were from multivariate logistic regression.

^a^Adjusted sex, age, current smoking, current drinking, BMI, SBP, TC, TG, LDL-C, HR, HbA1c at base line, new onset hypertension, diabetes, dyslipidemia, overweight/obesity during follow-up for kidney function and sex, age, current smoking, current drinking, BMI, HR at base line, New onset hypertension, diabetes, dyslipidemia, overweight/obesity during follow-up for metabolic status.

**Table S2.** Association between groups of different 95th percentile cut-off points of hyperfiltration and metabolic status and outcomes in all participants.

|  | Increased baPWV | | |  | Increased PP | | |
| --- | --- | --- | --- | --- | --- | --- | --- |
|  | case/N (%) | OR (95% CI)^a^ | *p* |  | case/N (%) | OR (95% CI)^a^ | *p* |
| *Groups* |  |  |  |  |  |  |  |
| A0H0 | 13/554(2.3) | Reference |  |  | 23/554(4.2) | Reference |  |
| A1H0 | 44/1477(3) | 1.31(0.673,2.532) | 0.43 |  | 88/1477(6.0) | 1.58(0.919,2.7) | 0.098 |
| A0H1 | 1/20(5.0） | 2.60(0.28,24.157) | 0.4 |  | 1/46(5.0) | 0.60(0.041,8.792) | 0.712 |
| A1H1 | 3/82(3.7) | 2.07(0.559,7.691) | 0.276 |  | 6/82(7.3) | 2.84(1.034,7.803) | 0.043 |
| *Interaction indexes* |  |  |  |  |  |  |  |
| S |  | 0.56 |  |  |  | 10.34 |  |
| API |  | 40.28% |  |  |  | 58.52% |  |
| API' |  | -77.82% |  |  |  | 90.33% |  |
| RERI |  | -0.835 |  |  |  | 1.66 |  |

PP: pulse pressure; baPWV: brachial–ankle pulse wave velocity; S: the synergy index; API: attributable proportion of interaction. API’: pure attributable proportion of interaction; RERI: the relative excess risk due to interaction; A0/A1: normal metabolism/abnormal metabolism, H0/H1: normal filtration/hyperfiltration.

^a^OR (odds ratio) and 95% CI (confidence interval) were from multivariate logistic regression, in which sex, age, current smoking, current drinking, BMI, HR at base line, New onset hypertension, diabetes, dyslipidemia, overweight/obesity during follow-up were adjusted.

**Table S3.** Association between hyperfiltration/abnormal metabolism and outcomes in all participants.

|  | Increased baPWV | | |  | Increased PP | | |
| --- | --- | --- | --- | --- | --- | --- | --- |
|  | case/N (%) | OR (95% CI)^a^ | *p* |  | case/N (%) | OR (95% CI)^a^ | *p* |
| *Kidney function* |  |  |  |  |  |  |  |
| Normal filtration | 213/1923(11.1) | Reference |  |  | 187/1923(9.7) | Reference |  |
| Hyperfiltration | 25/210(11.9) | 0.94(0.38,2.34) | 0.9 |  | 21/210(10.0) | 1.59(0.83,3.03) | 0.164 |
| *Metabolic status* |  |  |  |  |  |  |  |
| Normal metabolism | 14/574(2.4) | Reference |  |  | 24/574(4.2) | Reference |  |
| Abnormal metabolism | 47/1559(3.0) | 0.92(0.48,1.78) | 0.808 |  | 94/1559(6.0) | 1.46(0.84,2.51) | 0.177 |

PP: pulse pressure; baPWV: brachial–ankle pulse wave velocity.

^a^OR (odds ratio) and 95% CI (confidence interval) were from multivariate logistic regression.

^a^Adjusted sex, age, current smoking, current drinking, BMI, SBP, TC, TG, LDL-C, HR, HbA1c at base line, new onset hypertension, diabetes, dyslipidemia, overweight/obesity during follow-up, baPWV and PP at baseline respectively for kidney function and sex, age, current smoking, current drinking, BMI, HR at base line, New onset hypertension, diabetes, dyslipidemia, overweight/obesity during follow-up, baPWV and PP at baseline respectively for metabolic status.

**Table S4.** Association between groups of different hyperfiltration and metabolic status and outcomes in all participants.

|  | Increased baPWV | | |  | Increased PP | | |
| --- | --- | --- | --- | --- | --- | --- | --- |
|  | case/N (%) | OR (95% CI)^a^ | *p* |  | case/N (%) | OR (95% CI)^a^ | *p* |
| *Groups* |  |  |  |  |  |  |  |
| A0H0 | 13/528(2.5) | Reference |  |  | 22/528(4.2) | Reference |  |
| A1H0 | 42/1395(3) | 0.84(0.42,1.68) | 0.629 |  | 81/1395(5.8) | 1.31(0.74,2.31) | 0.354 |
| A0H1 | 1/46(2.2） | 0.49(0.06,4.20) | 0.515 |  | 2/46(4.3) | 0.63(0.09,4.25) | 0.632 |
| A1H1 | 5/164(3) | 0.96(0.32,2.93) | 0.946 |  | 13/164(7.9) | 2.44(1.08,5.49) | 0.032 |
| *Interaction indexes* | |  |  |  |  |  |  |
| S |  | 0.06 |  |  |  | -24 |  |
| API |  | 65.63% |  |  |  | 61.48% |  |
| API' |  | -15.75% |  |  |  | 104.17% |  |
| RERI |  | 0.63 |  |  |  | 1.5 |  |

PP: pulse pressure; baPWV: brachial–ankle pulse wave velocity; S: the synergy index; API: attributable proportion of interaction. API’: pure attributable proportion of interaction; RERI: the relative excess risk due to interaction; A0/A1: normal metabolism/abnormal metabolism, H0/H1: normal filtration/hyperfiltration.

^a^OR (odds ratio) and 95% CI (confidence interval) were from multivariate logistic regression, in which sex, age, current smoking, current drinking, BMI, HR at base line, New onset hypertension, diabetes, dyslipidemia, overweight/obesity during follow-up, baPWV and PP at baseline respectively, were adjusted.

**Table S5.** Association between different hyperfiltration status and outcomes in all participants.

|  | Increased baPWV | | |  | Increased PP | | |
| --- | --- | --- | --- | --- | --- | --- | --- |
|  | case/N (%) | OR (95% CI)^a^ | *p* |  | case/N (%) | OR (95% CI)^a^ | *p* |
| *Groups by the recovery of Hyperfiltration* |  |  |  |  |  |  |  |
| The rest^b^ | 57/1966(2.9) | Reference |  |  | 108/1966(5.5) | Reference |  |
| Hyperfiltration at baseline but normal at follow-up | 4/167(2.4) | 0.79(0.27,2.33) | 0.673 |  | 10/167(6.0) | 1.36(0.65,2.87) | 0.413 |
| *Groups by Hyperfiltration at baseline and follow-up* |  |  |  |  |  |  |  |
| H00 | 53/1890(2.8) | Reference |  |  | 98/1890(5.2) | Reference |  |
| H10 | 4/167(2.4) | 0.791(0.27,2.317) | 0.668 |  | 10/167(6.0) | 1.489(0.711,3.118) | 0.292 |
| H01 | 2/33(6.1) | 2.296(0.446,11.81) | 0.32 |  | 5/33(15.2) | 3.263(0.923,11.527) | 0.066 |
| H11 | 2/43(4.7) | 1.464(0.302,7.095) | 0.636 |  | 5/43(11.6) | 2.506(0.766,8.2) | 0.129 |
| *P* for trend | <0.001 |  |  |  |  | 0.018 |  |

PP: pulse pressure; baPWV: brachial–ankle pulse wave velocity; H00: Normal filtration at baseline and follow-up; H10: Hyperfiltration at baseline and Normal filtration at follow-up; H01: Normal filtration at baseline and Hyperfiltration at follow-up; H11: Hyperfiltration at baseline and follow-up.

^a^OR (odds ratio) and 95% CI (confidence interval) were from multivariate logistic regression.

^a^Adjusted sex, age, current smoking, current drinking, BMI, SBP, TC, TG, LDL-C, HR, HbA1c at base line, new onset hypertension, diabetes, dyslipidemia, overweight/obesity during follow-up, baPWV and PP at baseline respectively.

^b^The rest refers to all participants except those who are Hyperfiltration at baseline but normal at follow-up.

**Table S6.** Association between hyperfiltration/abnormal metabolism and outcomes in participants with high baPWV or PP at baseline, respectively.

|  | Increased baPWV | | |  | Increased PP | | |
| --- | --- | --- | --- | --- | --- | --- | --- |
|  | case/N (%) | OR (95% CI)^a^ | *p* |  | case/N (%) | OR (95% CI)^a^ | *p* |
| *Kidney function* |  |  |  |  |  |  |  |
| Normal filtration | 22/148(14.9) | Reference |  |  | 22/155(14.2) | Reference |  |
| Hyperfiltration | 4/28(14.3) | 1.57(0.42,5.90) | 0.508 |  | 6/20(30.0) | 4.79(1.03,22.36) | 0.046 |
| *Metabolic status* |  |  |  |  |  |  |  |
| Normal metabolism | 4/29(13.8) | Reference |  |  | 4/35(11.4) | Reference |  |
| Abnormal metabolism | 22/147(15.0) | 1.21(0.29,5.17) | 0.792 |  | 24/140(17.1) | 3.39(0.56,20.55) | 0.184 |

Participants with high baseline baPWV (≥1623.93cm/s) refer to those who are above the increased baPWV threshold (1705.13cm/s) by a 5% increase in baseline level. Participants with high baseline PP (≥60.64cm/s) refer to those who are above the increased PP threshold (63.67mmHg) by a 5% increase in baseline level.

PP: pulse pressure; baPWV: brachial–ankle pulse wave velocity.

^a^OR (odds ratio) and 95% CI (confidence interval) were from multivariate logistic regression.

^a^Adjusted sex, age, current smoking, current drinking, BMI, SBP, TC, TG, LDL-C, HR, HbA1c at base line, new onset hypertension, diabetes, dyslipidemia, overweight/obesity during follow-up, baPWV and PP at baseline respectively, for kidney function and sex, age, current smoking, current drinking, BMI, HR at base line, New onset hypertension, diabetes, dyslipidemia, overweight/obesity during follow-up, baPWV and PP at baseline respectively, for metabolic status.

**Table S7.** Association between different hyperfiltration status and outcomes in participants with high baPWV or PP at baseline, respectively.

|  | Increased baPWV | | |  | Increased PP | | |
| --- | --- | --- | --- | --- | --- | --- | --- |
|  | case/N (%) | OR (95% CI)a | p |  | case/N (%) | OR (95% CI)a | p |
| *Groups* |  |  |  |  |  |  |  |
| A0H0 | 4/22(18.2) | Reference |  |  | 3/30(10.0) | Reference |  |
| A1H0 | 18/126(14.3) | 0.63(0.15,2.61) | 0.522 |  | 19/125(15.2) | 3.00(0.42,21.55) | 0.275 |
| A0H1 | NA | NA |  |  | 1/5(20.0) | 1.58(0.02,125.42) | 0.837 |
| A1H1 | 4/21(19.0) | 1.28(0.21,7.61) | 0.789 |  | 5/15(33.3) | 18.97(1.80,200.13) | 0.014 |
| *Interaction indexes* |  |  |  |  |  |  |  |
| S | NA |  |  |  |  | 6.96 |  |
| API | NA |  |  |  |  | 81.11% |  |
| API' | NA |  |  |  |  | 85.63% |  |
| RERI | NA |  |  |  |  | 15.39 |  |

Participants with high baseline baPWV (≥1623.93cm/s) refer to those who are above the increased baPWV threshold (1705.13cm/s) by a 5% increase in baseline level. Participants with high baseline PP (≥60.64cm/s) refer to those who are above the increased PP threshold (63.67mmHg) by a 5% increase in baseline level.

PP: pulse pressure; baPWV: brachial–ankle pulse wave velocity; S: the synergy index; API: attributable proportion of interaction. API’: pure attributable proportion of interaction; RERI: the relative excess risk due to interaction; A0/A1: normal metabolism/abnormal metabolism, H0/H1: normal filtration/hyperfiltration.

^a^OR (odds ratio) and 95% CI (confidence interval) were from multivariate logistic regression, in which sex, age, current smoking, current drinking, BMI, HR at base line, New onset hypertension, diabetes, dyslipidemia, overweight/obesity during follow-up, baPWV and PP at baseline respectively, were adjusted.

**Table S8.** The correlation between hyperfiltration/abnormal metabolism and baPWV/PP at follow-up in all participants in generalized linear model.

|  | baPWV at follow-up | | |  | PP at follow-up | | |
| --- | --- | --- | --- | --- | --- | --- | --- |
|  | β (95% CI)^a^ | *P* for Omnibus Test^b^ | *P* for β |  | β (95% CI)^a^ | *P* for Omnibus Test^b^ | *P* for β |
| *Kidney function* |  |  |  |  |  |  |  |
| Hyperfiltration | 0.001(-0.008,0.010) | 0.787 | 0.787 |  | 0.007(-0.006,0.020) | 0.286 | 0.286 |
| *Metabolic status* |  |  |  |  |  |  |  |
| Abnormal metabolism | 0.017(0.011,0.023) | <0.001 | <0.001 |  | 0.017(0.008,0.026) | 0.001 | <0.001 |
| *Interaction* |  |  |  |  |  |  |  |
| Abnormal metabolism * Hyperfiltration | 0.016(0.005,0.027) | <0.001 | 0.030 |  | 0.023(0.007,0.039) | 0.002 | 0.006 |

PP: pulse pressure; baPWV: brachial–ankle pulse wave velocity.

^a^β and 95% CI (confidence interval) were from generalized linear model.

^a^Adjusted sex, age, current smoking, current drinking, BMI, SBP, TC, TG, LDL-C, HR, HbA1c at base line, new onset hypertension, diabetes, dyslipidemia, overweight/obesity during follow-up, baPWV and PP at baseline respectively for kidney function and sex, age, current smoking, current drinking, BMI, HR at base line, New onset hypertension, diabetes, dyslipidemia, overweight/obesity during follow-up, baPWV and PP at baseline respectively for metabolic status and interaction.

^b^*P* for Omnibus Test <0.05 indicated that the model was statistically significant.
